# Observations of Ball-Lightning-Like Plasmoids Ejected from Silicon by Localized Microwaves

**DOI:** 10.3390/ma6094011

**Published:** 2013-09-11

**Authors:** Yehuda Meir, Eli Jerby, Zahava Barkay, Dana Ashkenazi, James Brian Mitchell, Theyencheri Narayanan, Noam Eliaz, Jean-Luc LeGarrec, Michael Sztucki, Oleg Meshcheryakov

**Affiliations:** 1 Faculty of Engineering, Tel Aviv University, Ramat Aviv 69978, Israel; E-Mails: yehudam1@post.tau.ac.il (Y.M.); dana@eng.tau.ac.il (D.A.); neliaz@eng.tau.ac.il (N.E.); 2 Wolfson Applied Materials Research Center, Tel Aviv University, Ramat Aviv 69978, Israel; E-Mail: barkay@post.tau.ac.il; 3 Institut de Physique de Rennes, UMR N 6251 du CNRS, Université de Rennes I, 35042, Rennes cedex, France; E-Mails: brian.mitchell@univ-rennes1.fr (J.B.M.); jean-luc.le-garrec@univ-rennes1.fr (J-L.L.); 4 European Synchrotron Radiation Facility, Grenoble, F-38043, France; E-Mails: narayan@esrf.fr (T.N.); michi@sztucki.de (M.S.); 5 Wing Ltd., Odessa, 65000, Ukraine; E-Mail: wing99@mail.ru

**Keywords:** ball lightning, plasmoids, microwave heating, localized microwaves, dusty plasma, complex plasma, atmospheric plasma, silicon spheres, nanoparticles

## Abstract

This paper presents experimental characterization of plasmoids (fireballs) obtained by directing localized microwave power (<1 kW at 2.45 GHz) onto a silicon-based substrate in a microwave cavity. The plasmoid emerges up from the hotspot created in the solid substrate into the air within the microwave cavity. The experimental diagnostics employed for the fireball characterization in this study include measurements of microwave scattering, optical spectroscopy, small-angle X-ray scattering (SAXS), scanning electron microscopy (SEM) and energy dispersive X-ray spectroscopy (EDS). Various characteristics of these plasmoids as dusty plasma are drawn by a theoretical analysis of the experimental observations. Aggregations of dust particles within the plasmoid are detected at nanometer and micrometer scales by both *in*-*situ* SAXS and *ex-situ* SEM measurements. The resemblance of these plasmoids to the natural ball-lightning (BL) phenomenon is discussed with regard to silicon nano-particle clustering and formation of slowly-oxidized silicon micro-spheres within the BL. Potential applications and practical derivatives of this study (e.g., direct conversion of solids to powders, material identification by breakdown spectroscopy (MIBS), thermite ignition, and combustion) are discussed.

## 1. Introduction

Fireball-like plasmoids can be ejected by localized microwaves from a variety of solid and liquid substrates, and maintained buoyant in the air at atmospheric pressure, as demonstrated in [1,2]. This effect can be considered as an extension, from gas to solid initiation, of the phenomena of plasmoids excited by microwaves in gaseous atmospheres, e.g., the microwave electrode discharge effect [3] and microwave plasma reactors [4]. Fireballs excited by microwaves in air [5,6,7] were studied also in the context of their similarity to ball-lightning (BL) phenomena [8] (occasionally observed during stormy weather, volcanic activity and earthquakes; BL events are described by eye witnesses as peculiar luminous bodies floating in the air, bouncing on the ground, rotating as tornados, and even passing through windows). The excitations of laboratory BL-like effects have also been demonstrated experimentally using high-voltage discharges on silicon [9,10,11] and in water [12].

The ejection of fireballs by localized microwaves from solid substrates and their buoyancy in air [1] has been demonstrated for various substrate materials, e.g., glass, germanium, silicon, and salty water [2], and also for copper (forming of a fire-column like plasma) [13]. Borosilicate-glass fireballs have also been investigated by small-angle X-ray scattering (SAXS) at the European Synchrotron Radiation Facility (ESRF). The SAXS measurements revealed the presence of nanoparticles with diameters and number densities of ~50 nm and ~10^15^ m^−3^, respectively, within these fireballs [14,15]. In view of the significant presence of dust grains compared to electrons and ions, these fireballs have been considered in this study as a complex (dusty) plasma [16,17]. In another study [18], oxide nanoparticles were synthesized via microwave plasma decomposition of the initial materials.

In the present study, we apply both *in-situ* and *ex-situ* methods in order to quantitatively characterize the properties of the plasmoids ejected by localized microwaves from silicon-based substrates. In addition to known particles at nanometer scales, the present SEM observations identify silica micro-spheres on the order of ~10 μm diameter and larger, aggregated within the microwave-excited fireball. The electron density, the excitation and rotational temperatures are estimated as well using microwave scattering, optical spectroscopy and SAXS analyses, combined. The resemblance of these plasmoids to the natural BL phenomenon is discussed, as well as potential applications.

## 2. Experimental Setup

**Figure 1:**
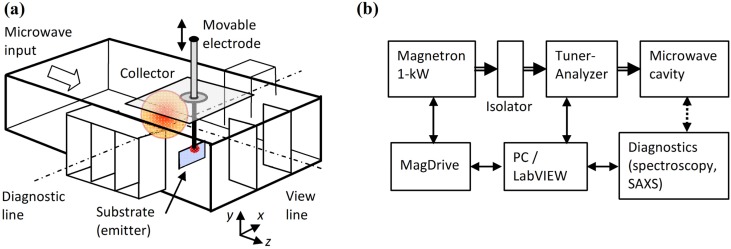
The experimental setup: (**a**) The microwave cavity with a movable electrode directing the localized microwaves into the substrate, thus creating a hotspot from which the fireball is ejected to the air atmosphere within the cavity; and (**b**) A block diagram of the experimental instrumentation.

The plasmoid investigated in this study is obtained by directing localized microwave power into a silicon-based substrate. A hotspot is created, from which the plasma is ejected and lifted up into the air atmosphere as a buoyant fireball within the microwave cavity [1]. The experimental setup depicted in Figure 1a,b consist of a microwave cavity made of a WR340 waveguide with additional openings (in microwave cutoff) for diagnostics, namely for the video imaging and optical spectroscopy line-of-sight, and for the synchrotron X-ray beam passing through the plasmoid (as in [1,13,14,15]). The substrate made of silicon is vertically positioned as in [19]. The movable electrode directs the microwave energy locally into the substrate.

The microwave power is generated by a 2.45-GHz, 1-kW magnetron unit, fed by a controllable switched-mode power supply (MagDrive-1000, Dipolar Ltd., Sydney, Australia). The microwave power is delivered to the chamber via an isolator and an impedance auto-tuner (Homer, S-Team Ltd., Singapore) as depicted in Figure 1b. The auto-tuner enables adaptive impedance matching and optimal transmission of microwave power to the fireball chamber, and it also provides real-time measurements of the complex impedance of the load.

**Figure 2:**
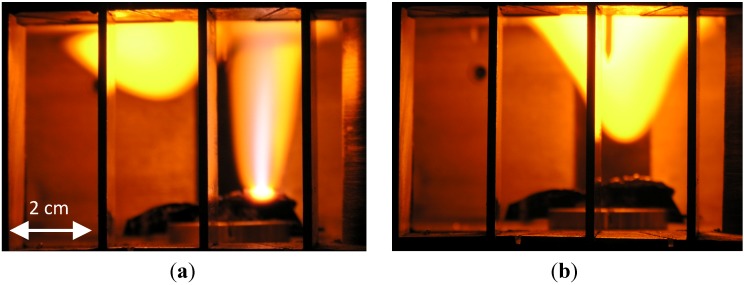
(**a**) A fire-column ejected from a hotspot created by localized microwaves in silicon, feeding an adjacent secondary fireball (plasmoid); and (**b**) The fireball evolved, floating in the air atmosphere within the microwave cavity [1].

The hotspot evolves at the contact point between the movable electrode and the substrate (Figure 1a) by a thermal-runaway process [20,21]. This localized-heating effect leads to melting of the substrate in a ~1 mm region near the electrode. Further heating of the hotspot results in evaporation and ionization of the substrate material. A fire column is ejected from the hotspot, and evolves as a buoyant luminous body floating stably in the air in a self-organized manner [1,22]. Typical images of a fire-column associated with a fireball and of a sole floating fireball are presented in Figure 2a,b, respectively.

A collector made of a copper plate, installed on the ceiling of the fireball chamber, accumulates the fireball dusty products. The other disposable components in each experiment include a silicon slice or a borosilicate glass rod used as a substrate (the emitter), and a tungsten or graphite rod used as an electrode. The fireball intensity can be enhanced by water-vapor inhalation (that can be enriched with sodium bicarbonate NaHCO_3_ [23,24]) sprayed as a salty aerosol into the microwave chamber.

The microwave-excited plasmoids are observed and measured by various diagnostic tools, including a microwave network analyzer (Homer, S-Team Lab, Bratislava, Slovak Republic), an optical spectrometer (Avaspec-3648, Avantes Ltd., Apeldoorn, the Netherland), SAXS, and scanning-electron microscopy (SEM). For the *in-situ* SAXS investigation, the fireball apparatus was installed at Beamline ID02 at the European Synchrotron Radiation Facility (ESRF), Grenoble, France. The surface morphology of the particles created by the fireball was characterized using a Quanta 200FEG (FEI Co., Hillsboro, OR, USA) environmental scanning electron microscope (ESEM). The ESEM was operated at high-vacuum and low vacuum modes using the Everhart-Thornley and the large field secondary electron detectors, respectively. The chemical element composition was analyzed using energy dispersive X-ray spectroscopy (EDS) with a Si(Li) liquid-nitrogen cooled Oxford INCA X-ray (Oxford Instrument, Oxfordshire, UK) detector.

## 3. Experimental Section

The experimental results for the plasmoid properties, obtained by microwave scattering, optical spectroscopy, SAXS, SEM and EDS measurements are presented and analyzed in the following sections. The variations in the microwave reflections during the process are analyzed using a dusty-plasma model and the other measurement results, as follows:

### 3.1. Microwave Scattering

**Figure 3:**
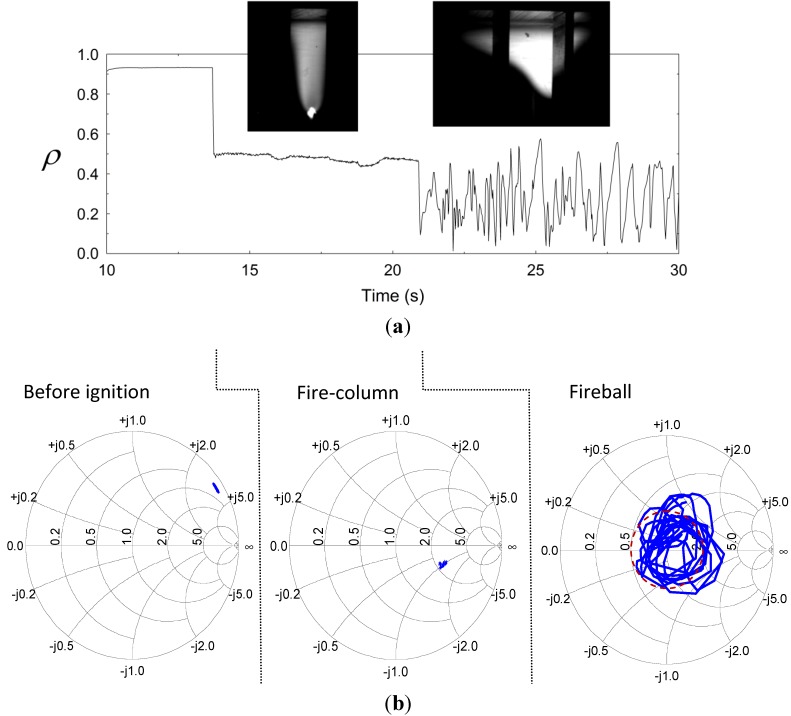
The microwave reflections from the cavity before and after ignition: (**a**) The autonomous reduction in the reflected power upon ignition of the fire-column, and its fluctuation in the fireball stage, being attracted by a minimal reflection level $\langle {\left|\rho \right|}^{2}\rangle \sim 0.1$; and (**b**) Smith-chart presentations of the cavity’s input impedance, before ignition, during the fire-column stage, and in the consequent fireball stage (the latter shows the tendency of the fireball to reach a self impedance matching).

A significant increase in the microwave power absorption at 2.45 GHz was observed after fireball ignition. This effect is displayed by the reduction of the reflection coefficient Γ=ρexp(jθ) in scalar and vectorial representations in Figure 3a,b, respectively. The microwave reflection shown in Figure 3a (in a scalar presentation) is significantly decreased at the time of ignition of the fire-column (from ρ ~ 0.9 to ~ 0.5). The reflection is decreased further at the fireball stage, while bouncing around ρ ~ 0.3 and attracted by the Smith-chart origin as shown in Figure 3b. The corresponding average power reflection, $\langle \rho^{2}\rangle$, is reduced to ~10% at this stage, hence the plasmoid tends to interact with its microwave generator as a nearly perfect load. Figure 3b presents a Smith-chart display [25] of this effect in vectorial plots of the reflected wave, before and after ignition. These results reveal the adaptive impedance-matching effect, which dictates the plasmoid’s autonomous evolution. The fluctuations observed in the fireball stage in Figure 3a,b are attracted by the origin of the Smith chart (at which ρ = 0) in an oscillatory manner. This self-tuning mechanism tends to adaptively maximize the microwave power absorbed by the fireball, by a self-tuning optimization of its intensity, position and size.

### 3.2. Optical Spectroscopy

**Figure 4:**
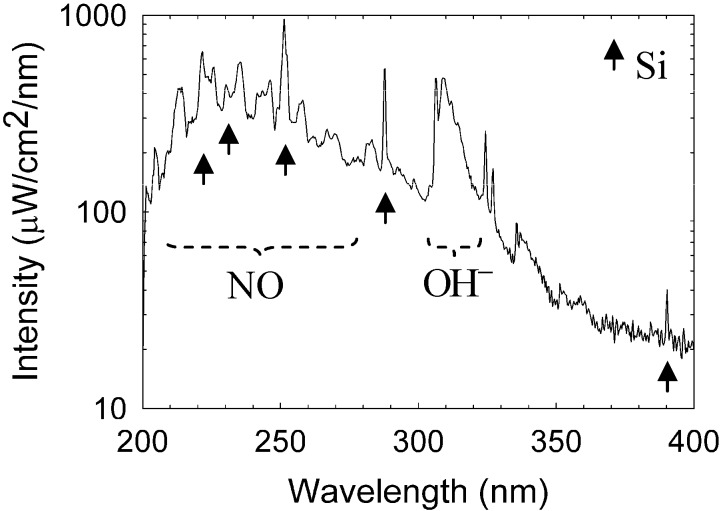
Optical spectroscopy measurements. A typical spectrum of silicon plasmoid reveals silicon excited atoms, and hydroxyl and nitric oxide radical production.

**Figure 5:**
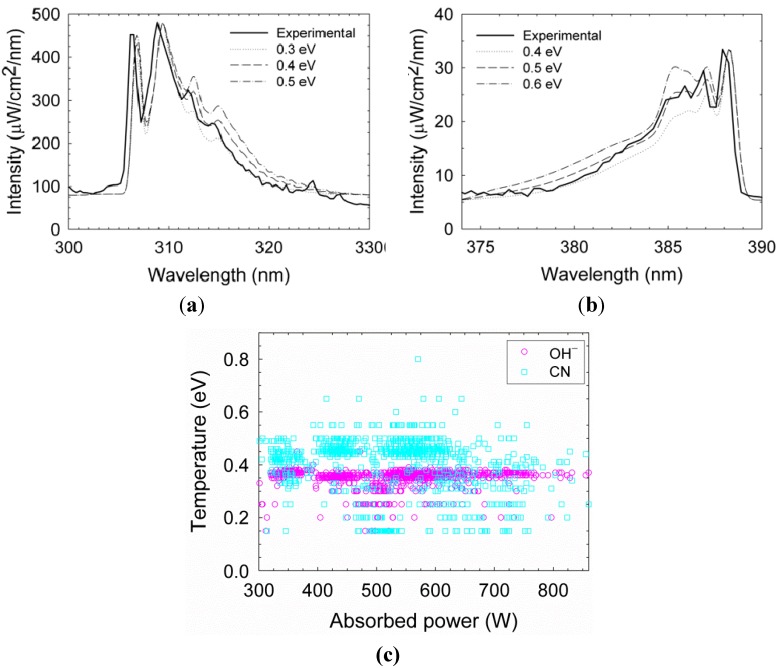
Temperature evaluation by fitting the radicals emission, (**a**) OH^–^; (**b**) CN by the LifBase simulation [27]; and (**c**) an accumulation of 941 fittings frames for OH^–^ and 973 frames for CN with respect to the instantaneous effective microwave power. The mean temperature does not seem to vary significantly with power in this range.

An Avaspec-3648 optical spectrometer was used to analyze the spectral emission of the plasmoid in the range of 200–1000 nm with a 0.3 nm resolution. The intensity is calibrated in this range by an AvaLight Deuterium-Halogen light source (DH-BAL-CAL UV/VIS, Avantes Ltd.). The experiment is conducted in humid air at atmospheric pressure, as describe in Section 2 above. The optical spectrum emitted by a typical plasmoid ejected from a silicon substrate is shown in Figure 4. Silicon lines are identified in the emission spectrum (in accordance with the substrate material from which the plasmoid has been ejected [26]). In addition, spectral emissions of nitric oxide (NO) and hydroxyl (${\text{OH}}^{-}$) radicals are also observed in Figure 4, hence these radicals are generated within the plasmoid (as observed also in copper fire-columns [13]). The ${\text{OH}}^{-}\;({\text{A}}^{2}{\text{Σ}}^{+}-{\text{X}}^{2}\text{Π})$ band emission in the range 300–330 nm reveals the rotational temperature of the radicals, estimated by fitting the line shape to the LifBase simulation [27]. As shown in Figure 5a, the ${\text{OH}}^{-}$ rotational temperature is estimated as ~0.4 eV. This result can be used also to evaluate the gas temperature within the plasmoid in some conditions [28,29]. The $\text{NO}({\text{A}}^{2}{\text{Σ}}_{\text{u}}-{\text{X}}^{2}\text{Π})$ gamma band shows the dissociation of water fumes as a result of combustion [30]. When using a graphite electrode, cyano (CN) radicals are also generated within the plasmoid. Their rotational temperature is estimated as ~0.5 eV by fitting to the LifBase simulation, as shown in Figure 5b. The violet spectrum of the CN band $\left({\text{B}}^{2}{\text{Σ}}^{+}-{\text{X}}^{2}{\text{Σ}}^{+}\right)$ can also be used to estimate the gas temperature [31,32]. An accumulation of 941 fittings frames for ${\text{OH}}^{-}$ and 973 frames for CN in various power levels, as shown in Figure 5c, results in mean temperatures of 0.34 ± 0.04 eV and 0.40 ± 0.11 eV for ${\text{OH}}^{-}$ and CN respectively. The data exhibit relatively stable temperatures, with no significant notable correlations with the absorbed microwave power.

**Figure 6:**
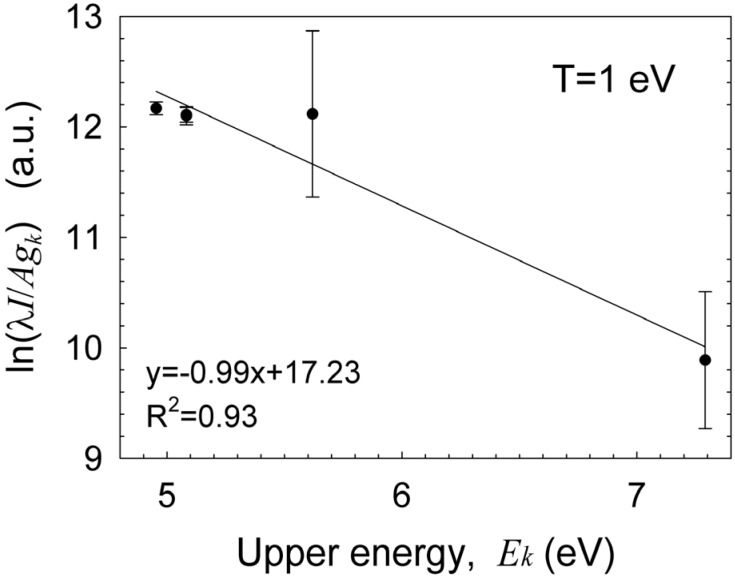
A Boltzmann plot of the silicon lines resulting in a ~1 eV excitation temperature.

**Figure 7:**
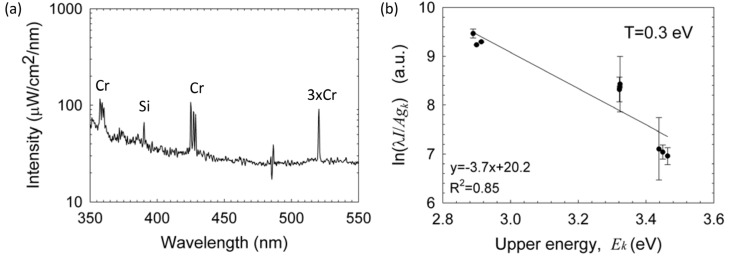
(**a**) An emission spectrum of silicon lines with chromium impurities; and (**b**) a Boltzmann-plot fit of the chromium lines resulting in ~0.3 eV excitation temperature. (Note a diode line defect at 485 nm in Figure 7a)

The Boltzmann plot technique [33] is applied to the silicon neutral lines, as shown in Figure 6, in order to estimate the excitation temperature. Assuming conditions for partial local thermal equilibrium (pLTE) as discussed below in Section 3.5, the Boltzmann equation for the intensity of the upper *k* to the lower *i* energy transition level; *I_ki_*, is given by $\text{ln}\left(I_{ki}\lambda_{ki}/g_{k}A_{ki}\right)=-\left(1/k_{B}T_{exc}\right)E_{k}+const.$; where λ*_ki_* and *A_ki_*, are the wavelength and transition probability, respectively; *k_B_* is the Boltzmann constant; and *E_k_* is the upper energy state with a degeneracy *g_k_*. Therefore, the slope of the curve obtained is inversely proportional to the plasma excitation temperature, *T_exc_*. The results show an excitation temperature of ~1 eV for the detected silicon lines. Impurities originating probably from the electrode are also observed, e.g., the chromium emission lines marked in Figure 7a. Similarly to the analysis of radical molecules presented above, the Boltzmann-plot technique applied to the chromium spectral lines results in ~0.3 eV as shown in Figure 7b (note that this plot may be more accurate than that for silicon in Figure 6 due to the larger number of chromium spectral lines, the partial overlap of the silicon lines and the NO radical emission, and the relatively low spectral resolution and sampling available).

### 3.3. SAXS Measurements

**Figure 8:**
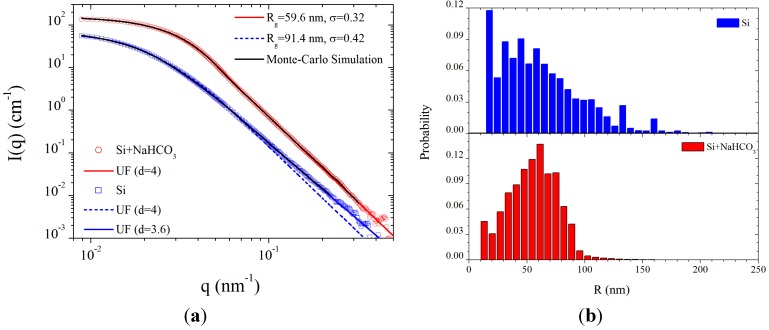
(**a**) Normalized SAXS intensity profiles from the silicon fireball ejected by a graphite electrode in dry air and in damp air with NaHCO_3_ (Blue and Red curves, respectively), together with model fits; and (**b**) Particle size distribution derived from the best fit Monte-Carlo simulations corresponding to mean particle radii of 62.9 nm and 55.4 nm for the dry and damp conditions, respectively.

The fireball apparatus was installed at the ESRF for *in-situ* SAXS measurements and the X-ray beam was aligned along the diagnostic line illustrated in Figure 1. X-ray scattering experiments were performed by illuminating the fireball with a 12.4 keV X-ray (λ ~ 0.1 nm) and detecting the scattered X-rays using a high brilliance pinhole SAXS instrument. For comparison, the SAXS measurements were performed both in a dry air atmosphere and in damp air enriched with a micro-droplet aerosol of water solution of NaHCO_3_ (decomposed above 70 °C into sodium carbonate, water and carbon dioxide. The carbonate converts at ~1000 °C to sodium oxide ${\text{Na}}_{2}{\text{CO}}_{3}\to {\text{Na}}_{2}\text{O}+{\text{CO}}_{2}$ [34]). Representative SAXS profiles are shown in Figure 8a as a function of the scattering vector

(1) q=(4π/λ)sin(θ/2)

where *θ* and λ are the scattering angle and X-ray wavelength, respectively. It should be noted that these intensities correspond to their maximum level while scanning the fireball apparatus through the X-ray beam, and therefore represent the region relatively concentrated with particles. The SAXS intensities from the dry air fireball were found to be relatively stable, while some level of intermittency was observed during the damp-air fireball lifetime.

The SAXS data are modeled in terms of a single level unified scattering (UF) function [35], in the form

(2) $$I\left(q\right)=G\text{ exp}\left(-q^{2}R_{g}^{2}/3\right)+B{\left({q/\left[\text{erf}\left(qR_{g}/\sqrt{6}\right)\right]}^{3}\right)}^{-d}$$

as in [13], where the whole scattering curve could be described by a single structural level with four parameters: radius of gyration (*R_g_*); amplitudes *G* and *B* of the exponential and power law terms, respectively; and power law exponent (*d*). The best-fit parameters yielded the *R_g_* and standard deviation (σ) as indicated in the upper legend of Figure 8a. In addition, a comparison with the scattering profiles obtained by Monte-Carlo simulation method [36] using 50,000 particles is also shown. The two plots in Figure 8b show the size distribution derived from the Monte-Carlo simulation method [36]. Evidently, these size distributions are more complex than typical log-normal distribution assumed by the unified analysis [35]. The mean particle radii from the simulations are 62.9 nm and 55.4 nm for the dry and damp conditions, respectively.

From the unified analysis, the best fit value of *d* for the dry air fireball (*d* = 3.6) is smaller than the Porod law (*d* = 4), suggesting that the particles have a rough fractal surface characterized by a surface fractal dimension of 2.4 (*d* − 6). However, in the case of the damp-air fireball, the data clearly display a Porod behavior (*d* = 4) indicating a smooth particle-air interface. The intermittency in this case implies that both smaller and larger particles were present. The smooth interface and narrower spread of the size distribution suggests that particles under this condition may be in the form of molten droplets. These droplets could coalesce and form the larger silica spheres observed intermittently in the SAXS analysis and seen also in the SEM images presented below.

Assuming a melt density of Si (2570 kg/m^3^), the particle number densities in the relatively concentrated region are ~10^15^–10^16^ m^−3^ for both fireball types (compared to ~10^15^ m^−3^ found in fireballs generated from glass substrates [13]). The size range deduced from in-situ SAXS is consistent with the ex-situ SEM observations. For the mean radii derived from simulations, *R_m_* ≈ 60 nm, the maximum particle volume fraction is ~10^−6^ within the fireball (as the SAXS analysis was performed using the highest levels of intensities).

### 3.4. SEM Observations

**Figure 9:**
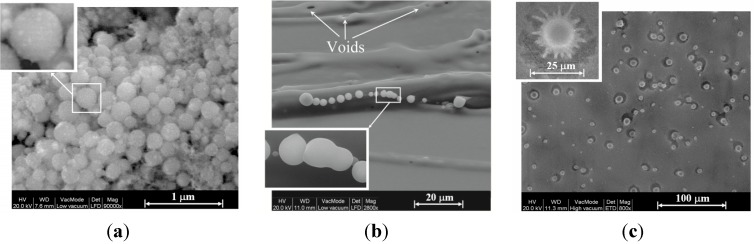
SEM observations: (**a**) aggregates of spherical particles, 0.1–0.3 μm in diameter, on the silicon emitter. The inset shows a typical sphere with a rough hairy surface that consists of smaller particles, ~10 nm in diameter. EDS analysis reveals mostly silicon and oxygen in these aggregates; (**b**) larger spheres, on the micrometer-scale in diameter, in a molten region, and voids of similar sizes; and (**c**) micro-spheres that seem to be smashed onto the copper collector plate. The inset shows a typical ~10 μm sphere of silicon that seemingly was crushed into the copper plate and has created a crater by hitting the surface.

The SEM analysis was restricted to samples soon after microwave preparation without any modification or coating. The fireballs generated in these conditions create spherical particles of various sizes and surface roughness, including particles larger than the SAXS detection limit (~120 nm) in our experiment. Figure 9a shows aggregates of such particles observed by SEM on the silicon emitter (the experimental setup consisted of a Si emitter, Cu collector, and C electrode, in a water-vapor sprayed atmosphere). The sub-micron spheres obtained are characterized by a rough surface, which consists of smaller particles, of the order of ~10 nm diameter. As revealed by EDS analyses, these aggregates are mostly made of silicon and oxygen. Larger spheres of several micrometers in diameter are observed in the molten region of the silicon emitters, for instance the string of micro-spheres shown in Figure 9b. The molten surface also includes voids of similar sizes (which may indicate the origin of micro-particles that bubbled out and were ejected from the surface). Figure 9c shows a SEM image of micro-spheres that seem to be sputtered and crushed onto the ceiling of the collector. They reside in craters that could be created by their collisions with the copper collector surface.

**Figure 10:**
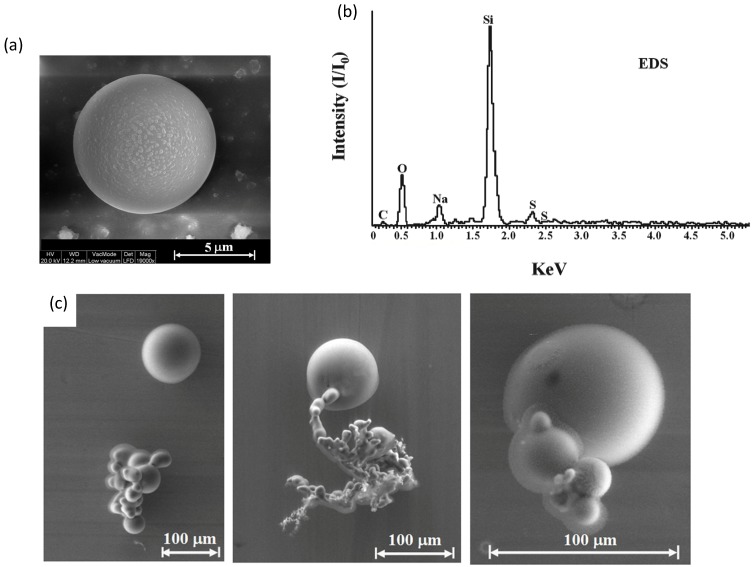
A typical perfect sphere, 8 μm in diameter; (**a**) the SEM image; (**b**) its EDS spectrum showing mostly Si and O; and (**c**) examples of micro-particles observed by SEM on the copper collector as clusters in various stages of agglomeration.

An SEM image of a perfectly round sphere of 8 μm in diameter, typically observed on the collector ceiling, is shown in Figure 10a. The sub-micron particles on its outer surface may indicate that the formation of micrometer-scale spheres involves smaller particles’ agglomeration. The EDS analysis of perfect spheres mostly show Si and also O to a lesser extent, as in Figure 10b, which may indicate surface oxidation of the bulk silicon sphere. Minor impurities of other elements, including C, Na and S, were also detected by the EDS analysis of the perfectly round sphere. The consolidation of nanoparticles to larger aggregates melted together is seen in various examples in Figure 10c. The larger particles observed in this experiment, mostly on the upper collector, are slightly oxidized silicon spheres on the order of ~10 μm and larger.

The EDS analyses of these spheres show the presence of Si as well as O, with small amounts of other elements including Cl, C and Cu. The smaller particles, up to ~80 nm in diameter, are also observed on the silicon emitter and *in-situ* by SAXS. Hence, one may deduce that the larger spheres agglomerate within the fireball while being elevated up to the collector. In view of these SEM images, one may deduce that some of these large spheres have evolved as hollow spheres with a ~1 μm thick shell, as seen by their crushed traces on the collector (Figure 9c).

**Figure 11:**
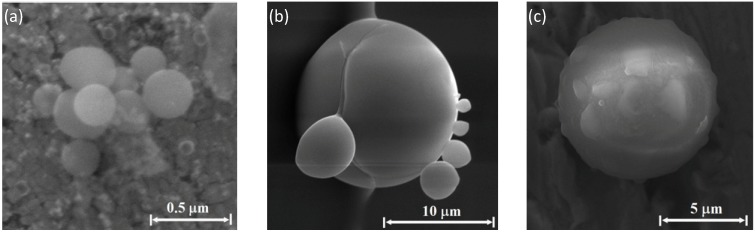
The particles presented were created by a Si-emitter, C-electrode, and Cu-collector system, in water-vapor sprayed air atmosphere: (**a**) examples of micro-particle clusters observed by SEM on the silicon emitter; (**b**) a micro-particle cluster observed by SEM on the silicon emitter; and (**c**) around sphere obtained in the same conditions with sodium bicarbonate added to the water vapor inhalator.

**Figure 12:**
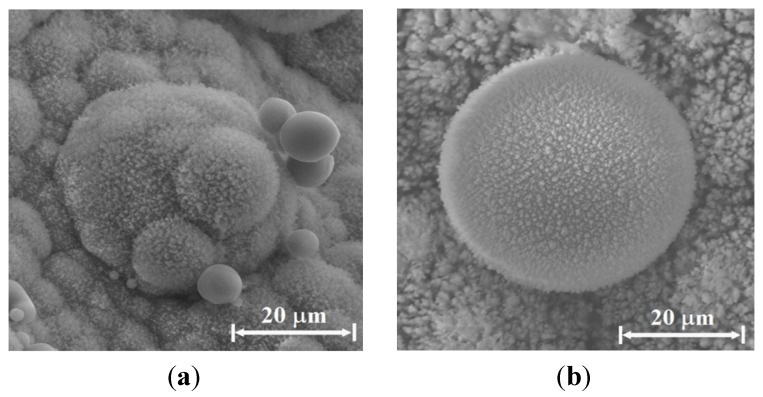
Examples of hairy sea-urchin-like clusters observed by SEM on the copper collector, as (**a**) was created by a Si-emitter, C-electrode, and Cu-collector system, in water-vapor sprayed air atmosphere; and (**b**) was obtained in the same conditions with sodium bicarbonate added to the water vapor inhalator.

The consolidation of nanoparticles to larger aggregates melted together is seen in various examples of clusters in various stages of agglomeration in Figure 11 and Figure 12. Figure 11a–c show particles observed on the Si emitter. A cluster of sub-micron spherical particles (~0.2 μm diameter each) mostly made of silicon and oxygen is shown in Figure 11a, whereas Figure 11b presents a larger sphere (~15 μm diameter) with several smaller spheres attached to it. Adding sodium bicarbonate (NaHCO_3_) to the water sprayed vapor results in round spheres, as the ~10 μm diameter sphere presented in Figure 11c. The latter is mostly made of silicon and oxygen, with smaller quantities of carbon. Figure 12a,b respectively show larger sea-urchin like particles (~40 μm) observed on the copper collector surface, without and with sodium bicarbonate enrichment of the water sprayed vapor. The accumulation of smaller particles over big round particles is seen both in Figure 11b and in Figure 12a under the same conditions.

Similar structures with the same elemental composition were also observed on the C electrode surface. In addition to Si and O, the EDS and optical spectroscopy show traces of Na, Ca, Fe, K, Zn, P, Al and Ti, as in other silicon breakdowns in air [37]. The silicon micro-spheres observed in this study resemble those produced by flames [38], microwave plasmas [15], short-pulse lasers [39], and thermal evaporation techniques [40]. Future SEM-EDS study would include cross-section samples for depth chemical composition profile.

### 3.5. Analysis

The microwave reflections measured at the input port of the cavity enable us to estimate the effective impedance of the plasmoid, and, consequently, to find its relative dielectric permittivity, *ε_r_*, the latter consists of the complex plasma permittivity $\varepsilon_{p}={\varepsilon^{\prime }}_{p}-j{\varepsilon^{\prime \prime }}_{p}$ and the dust conductivity, σ*_ed_*, thus the total effective permittivity is given by

(3) $$\varepsilon_{r}=\varepsilon_{p}-j\sigma_{ed}/\varepsilon_{0}\omega$$

where *ε_0_* is the vacuum permittivity; and *ω* is the angular frequency. The dielectric permittivity of the plasma is given by its real and imaginary components, ${\varepsilon^{\prime }}_{p}=1-\omega_{p}^{2}/\left(\omega^{2}+\upsilon^{2}\right)$ and ${\varepsilon^{\prime \prime }}_{p}=\omega_{p}^{2}\upsilon /\omega (\omega^{2}+\upsilon^{2})$, respectively, where $\omega_{p}=\sqrt{e^{2}n_{e}/m_{e}\varepsilon_{0}}$ and *υ* are the plasma and collision frequencies, respectively. The dusty plasma conductivity is given in similar conditions by [41] as

(4) $$\sigma_{ed}≅\eta_{ed}\frac{\omega }{k}\left[\frac{\omega^{2}-\upsilon \upsilon_{ch}}{\left(\omega^{2}+\upsilon_{ch}^{2})(\omega^{2}+\upsilon^{2}\right)}+j\omega \frac{\upsilon +\upsilon_{ch}}{\left(\omega^{2}+\upsilon_{ch}^{2})(\omega^{2}+\upsilon^{2}\right)}\right]$$

where $\eta_{ed}=n_{e}n_{d}\text{π}r_{d}^{2}e^{2}/m_{e}$ is the charging factor; $n_{e}$ and $n_{d}$ are the electron and dust grain densities, respectively; $r_{d}$ is the average dust particle radius; e and $m_{e}$ are the electron charge and mass, respectively; and *k* is the wave wavenumber. The electron effective collision frequency is given by $\upsilon ={\text{V}}_{\text{Te}}\sigma_{n}N_{n}$, where ${\text{V}}_{\text{Te}}=\sqrt{kT_{e}/m_{e}}$ is the electron thermal velocity; σ*_n_* and *N_n_* are the neutrals cross-section and density, respectively; *T_e_* is the electron temperature; and *υ_ch_* is the dust charging frequency. Assuming $\upsilon \upsilon_{ch}<<\omega^{2}$ and $\upsilon_{ch}<<\omega$, and substituting Equation (4) into Equation (3), the latter is further reduced to

(5) $$\varepsilon_{r}≅1+\frac{\omega_{p}^{2}}{\omega^{2}+\upsilon^{2}}\left[-1+\frac{\upsilon }{kl_{d}\omega }-j\left(\frac{\upsilon }{\omega }+\frac{1}{kl_{d}}\right)\right]g$$

where *k* is approximated by the axial wavenumber in the waveguide; *l_d_* is defined here as the mean free path for electron-dust collisions,

(6) $$l_{d}=\frac{1}{\pi r_{d}^{2}n_{d}}$$

and *g* is a spatial profile function for the plasmoid in the waveguide.

**Figure 13:**
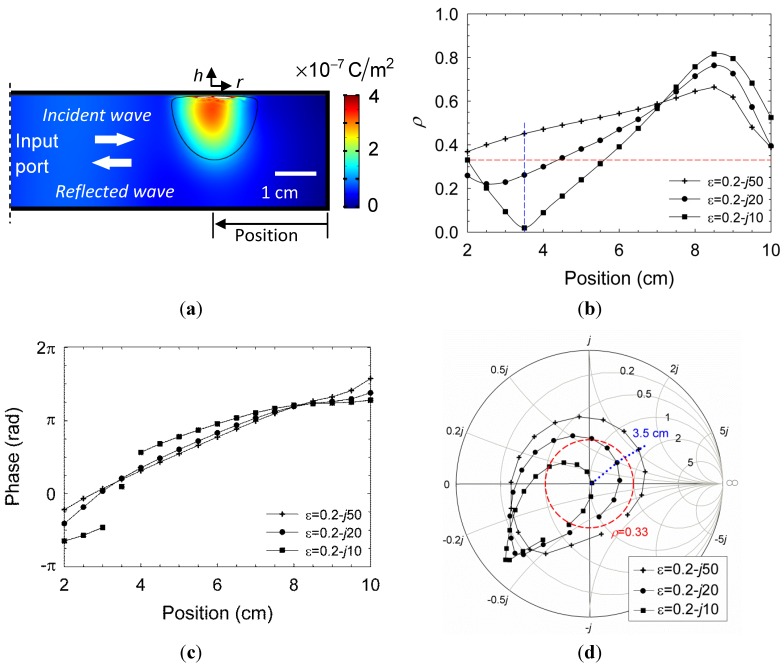
A parametric estimation of the dusty plasmoid properties by the microwave reflection measurements: (**a**) The plasmoid shape and position used in the numerical model presented by the amplitude profile of the displacement field for *ε_r_* = 0.2 − *j*20; (**b,c**) The amplitude and phase, respectively, of the reflection coefficient as a function of the plasmoid position, for various values of *ε_r_*; and (**d**) A Smith-chart presentation for the plasmoid’s impedance. The dashed Red circle indicates the self-impedance matching region (|Γ|≤0.33) as observed experimentally in Figure 3b. The dotted Blue curve marks the 35 mm position at which a perfect match is obtained for *ε_r_* = 0.2 – *j*10.

The model illustrated in Figure 13a is employed using COMSOL MultiPhysics^TM^ in order to estimate the corresponding permittivity of the plasmoid, ε*_r_*, given the measurements of the reflection coefficient, Γ, as in Figure 3b. A Gaussian profile is taken for the plasmoid, similarly to Figure 2b, with a ~2 cm core diameter (*i.e.*, $g\propto \text{exp}\left(-r^{2}/w_{r}^{2}-h^{2}/w_{h}^{2}\right)$ where *r* and *h* are the plasmoid’s cylindrical coordinates, illustrated in Figure 13a, and *w_r_* = 1 cm and *w_h_* = 1.6 cm are the corresponding radii of the plasmoid’s core in the radial and axial dimensions). The model also assumes a ~1 mm sheath barrier isolating the plasmoid from the waveguide ceiling. The complex ε*r* space is scanned numerically by the simulation in order to find the conditions that satisfy |Γ|≤0.33 as observed in the experiments.

The amplitude profile of the displacement field **D** = *ε_0_ε_r_***E** along the waveguide is presented for instance in Figure 13a for *ε_r_* = 0.2 − *j*20 (in which the reflections attain |Γ|≤0.33). The corresponding reflection coefficients, amplitude and phase, are shown in Figure 13b,c, respectively, as functions of the axial position (*i.e.*, the distance from the end mirror as indicated in Figure 13a) for several values of *ε_r_* A nearly perfect impedance matching (no reflection) is observed for *ε_r_* = 0.2 – *j*10 at an axial position of 35 mm. Other positions or higher dielectric losses of the plasmoid decrease its microwave absorption. This effect is illustrated also by the Smith-chart presentation in Figure 13d, which maps the complex relation between *ε_r_* and the microwave reflection coefficient Γ in various positions. The area within the dashed circle in Red marks the |Γ|≤0.33 region, with less than 10% microwave power reflection. A longitudinal periodicity of about half a wavelength is notable in Figure 13b,c,d.

The Boltzmann-plot analysis applied under the near-LTE assumption [33] to the spectra measured in Section 3.2 results in electron temperatures in the range of 0.3–1.0 eV. The corresponding electron thermal velocity is *V_Te_* ~ 2.3 × 10^5^ ms^−1^ hence the collision frequency is υ ~ 10^10^ s^−1^ (assuming σ*_n_* ~ 4.4 × 10^−20^ m^2^ and *N_n_* ~ 10^24^ m^−3^ for a weakly ionized dusty plasma in air atmosphere [41]). The dust size and number densities given by the SAXS measurements enable us to deduce the dust conductivity component in *ε_r_* (3), and hence to estimate the electron density by the remaining *ε_p_* components.

The SAXS measurements in Section 3.3 yield dust density and size distributions with mean values in the order *n_d_* ~ 10^15^ m−^3^ and *r_d_* ~ 60 nm, respectively, for dry air hence the mean free path for electron-dust collisions (6) is *l_d_* ~ 10^−2^ m. In these conditions, for ω ≈ 1.6 × 10^10^ s^−1^, the real part in Equation (5), $-1+\upsilon /kl_{d}\omega ∼0$, is significantly smaller than the imaginary part, which plays the dominant role in the microwave absorption by the plasmoid. Hence, the plasma frequency results in $\omega_{p}\sim 10^{11}$ s^−1^ for *ε_r_* = 0.2 − *j*20, and the corresponding electron density is estimated as *n_e_* ~ 3 × 10^18^ m^−^^3^ in this case.

The resulting electron density is slightly lower than the limit derived by Griem’s criterion [33] for LTE. However, it may satisfy the partial-LTE (pLTE) condition [42]. In atmospheric-pressure plasmas, the LTE assumption may be valid in the core of the plasmoid, but not on its periphery [43]. The equilibrium may exist since the higher pressure in the core increases the collision rate between the electrons and heavy particles, hence the energy transfer becomes more effective for a given electron density. The contributions of the electron and dust components to the total value of *ε_r_* in Equation (5) are comparable; hence the dust particles play a non-negligible role in the microwave absorption by the plasmoid.

The fireball attraction to the Smith-chart origin is observed experimentally as the self-impedance-matching effect presented in Section 3.1 (Figure 3b). This is an oscillatory effect since once the fireball is perfectly matched, more power is absorbed, which increases the electron density (e.g., along the dotted blue curve in Figure 13d. The consequent increase in the plasmoid conductivity violates the perfect matching condition; hence the system is bouncing back and forth around its equilibrium state, as was also observed experimentally.

## 4. Discussion

The generation of silicon nano- and micro-spheres by BL-like plasmoids in this experiment partly mimics the natural BL mechanism, as modeled for instance in [44,45]. This model suggests that BL is generated by a normal lightning bolt that strikes the ground and evaporates silica and carbon, and that the BL luminosity is maintained by a slow combustion process in which silicon nanoparticles are gradually fused together to form larger silicon spheres with oxidized outer surfaces. According to this model, reactions like

SiO_2_ + 2C → Si + 2CO (7)

reduce SiO_2_ to silicon in the form of nanoparticles. The BL evolves as a stable and elastic aerogel structure, as a result of mutual electrostatic aggregation of the bipolar charged Si, SiO or SiC based aerosol nanoparticles. Due to atmospheric oxidation, silicon dioxide layers grow on the surface of these nanoparticles (e.g., Si + O_2_ → SiO_2_). These passivating oxide surface layers significantly reduce air oxygen diffusion to the silicon cores of the nanoparticles and hence reduce the rate of the oxidation of nanoparticles [46].

Another model [23,24] suggests that BL is an electromagnetically self-compressed cloud of unipolar charged combustible aerosol particles, e.g., such as metallic (or metalloid-based) nanoparticles, whose oxidation is inhibited by electrolyte surface layers consisting of metal hydroxides, oxy-hydroxides or hydroxo-carbonates, which normally grow on the metal nanoparticle surface in the course of its oxidation in standard humid air at relatively low temperatures.

In this experiment, the microwave energy is directed by a graphite electrode, imitating the lightning bolt strike, and creates a molten hotspot in the solid silicon substrate from which the fireball is ejected. The role of the initiating normal lightning is played here to some extent by the localized microwave energy [47]. The fireball intensity is enhanced by the humidity in a damp air atmosphere [24], and even further if enriched by salty aerosols of sodium bicarbonate NaHCO_3_ (baking soda). The salty damp air atmosphere also mimics the stormy weather conditions typical of BL appearance, particularly near sea shores. The formation of BL-like objects from silicon substrates in the present experiments is possibly enhanced also by the exothermic electrochemical mechanism of the slow oxidation of the charged hydrated silicon nanoparticles. This slow oxidation may occur also through the dynamic porous volatile layers of the mixed silicic acids growing on the hydrated nanoparticle surface in such an electrolyte-phlegmatized ion-mediated oxidative process (Si + O_2 _+ nH_2_O → [SiO_x _(OH)_4-2x_]_n_+ at least 911 kJ). The small addition of an alkaline salt in this experiment, catalyzes the reaction between silicon and water, and slightly changes the geometrical features of the observed particles. In addition to water vapor induced formation of the passivating surface electrolyte layers [24], the presence of alkali or alkali-earth metal oxides in many soil silicates could also contribute to the formation of molten silicate-based electrolyte shells on the surface of co-condensed silicon nanoparticles [23]. This contribution would take place in a slightly modified model for the spontaneous generation of an ensemble of the silicon/air aerosol or aerogel nano-batteries. Thus, according to [23,24], the natural formation of the surface electrolyte layers converts the oxidizable metal aerosol nanoparticles into aerosol metal/air core-shell nano-batteries, periodically short-circuited by electron field or thermionic emission. This predominantly electrochemical, ion-mediated oxidation of charged combustible nanoparticles in humid air helps to explain the intense electromagnetic dipolar attraction between them, *i.e.*, cohesion of the BL aerosol substance, as observed.

In both current models [23,44,45] that use slowed oxidation of combustible nanoparticles to explain BL, processes of possibly repeated fusions of separate charged metal nanoparticles with the final formation of micrometer-sized particles could also considerably slow down their complete oxidation due to a substantial decrease of the overall surface area. Thus, micrometer-sized metal or metalloid combustible particles produced by repeated coalescence of the initially nano-sized particles could be even more probable as long-lived solid fuel component of the BL material.

It is noted that the estimated value of the electron density found in Section 3.5 might be lower than expected. The deviation may possibly stem from the different averaging scales of the various measurements combined in this analysis. While the microwave reflection is volumetrically averaged, the spectral and SAXS measurements collect signals along their line-of-sight axes only. Furthermore, the optical spectrum is emitted mostly from the plasma core, whereas the SAXS measurements accumulate signals from particles in the outer layers of the fireball. Such differences in the spatial and temporal averaging scales of the various parameters measured may bias the electron-density estimate. Further studies are still needed to elaborate this analysis.

## 5. Conclusions

Various experimental techniques have been employed in this study in an attempt to characterize the microwave-excited plasmoid ejected by localized microwaves from silicon-based substrates in atmospheric pressure environment. Each of these methods is limited in certain aspects, but their ensemble provides a better insight of the fireball properties.

The fireball studied here is characterized as a partially ionized complex (dusty) plasma, as indicated by the low electron density calculated (*n_e_* ~ 3 × 10^18^ m^−3^), the low temperatures measured (<1 eV), the lack of spectral atomic ionization lines, and the presence of nanoparticles (*n_d_* ~ 10^15^ m^−3^) forming the dusty plasmoid. These particles made of the substrate material seem to agglomerate into larger structures in the micrometer scale. The effect of the fireball’s self-impedance matching tends to maximize the absorbed power and to maintain its buoyancy. The dielectric permittivity of the buoyant plasmoid is attributed both to the free electrons and to the charged dust particles. The chemical reactions and combustion processes are evidenced by the radical production within the plasmoid. These laboratory fireballs resemble natural BL, also in the aspects of aggregation and oxidation of silicon micro-particles (e.g., [44,45]).

These findings, although requiring further studies, may advance the multi-disciplinary understanding of the BL enigma. Beside the scientific aspects of the plasmoid and BL studies, derivatives of this work are considered for applications, such as direct conversion of solids to powders in air atmosphere [13], microwave induced breakdown spectroscopy (MIBS) for material identification [26], ignition of thermite reactions [48], environmental applications of the fireball UV and radical emission [29], developments of nano-battery concepts [23], micro-sphere production techniques (e.g., for optical applications [49]), and microwave enhanced combustion processes.
